# Improvement of the Flavanol Profile and the Antioxidant Capacity of Chocolate Using a Phenolic Rich Cocoa Powder

**DOI:** 10.3390/foods9020189

**Published:** 2020-02-14

**Authors:** Rocío González-Barrio, Vanesa Nuñez-Gomez, Elena Cienfuegos-Jovellanos, Francisco Javier García-Alonso, Mª Jesús Periago-Castón

**Affiliations:** Department of Food Technology, Food Science and Nutrition, Faculty of Veterinary Sciences, Regional Campus of International Excellence “Campus Mare Nostrum”, Biomedical Research Institute of Murcia (IMIB-Arrixaca-UMU), University Clinical Hospital “Virgen de la Arrixaca”, University of Murcia, Espinardo, 30100 Murcia, Spain; vanesa.nunez@um.es (V.N.-G.); elena.cienfuegosjovellanos@gmail.com (E.C.-J.); fjgarcia@um.es (F.J.G.-A.); mjperi@um.es (M.J.P.-C.)

**Keywords:** phenolic compounds, HPLC-DAD, fluorescence detection, flavan-3-ols, procyanidins, ORAC, (+)-catechin, (−)-epicatechin, dark chocolate

## Abstract

Chocolate is made from cocoa, which is rich in (poly)phenols that have a high antioxidant capacity and are associated with the prevention of chronic diseases. In this study, a new production process was evaluated in order to obtain a dark chocolate enriched in (poly)phenols using a cocoa powder with an improved flavanol profile. The antioxidant capacity (Oxygen Radical Absorbance Capacity (ORAC) assay) and the flavanol profile (HPLC-DAD and HPLC-FL) was determined. The analysis of the enriched chocolate showed that the total flavan-3-ols (monomers) content was 4 mg/g representing a 3-fold higher than that quantified in the conventional one. Total levels of dimers (procyanidin B1 and B2) were 2.4-fold higher in the enriched chocolate than in the conventional, with a total content of 6 mg/g. The total flavanol content (flavan-3-ols and procyanidins) in the enriched chocolate was increased by 39% compared to the conventional one which led to a 56% increase in the antioxidant capacity. The new flavanol-enriched dark chocolate is expected to provide greater beneficial effect to consumers. Moreover, the amount of flavonols provided by a single dose (ca. 200 mg per 10 g) would allow the use of a health claim on cardiovascular function, a fact of interest for the cocoa industry.

## 1. Introduction

In recent years, the consumption of cocoa and cocoa products has aroused greater interest due to their beneficial health effects. Cocoa beans and their derivate products are rich in (poly)phenols, which are associated with the prevention of diseases related to oxidative stress, such as cardiovascular diseases, carcinogenic processes, and neurodegenerative diseases [1,2,3]. However, the beneficial effects of cocoa (poly)phenols depend on the amount consumed, their bioavailability, and the biological activity of the conjugates formed [4]. Cocoa contains several classes of phenolic compounds among which, flavan-3-ols, procyanidins and anthocyanins [5]. Flavanols (flavan-3-ols and procyanidins) are the most studied compounds in cocoa and its derivatives for their beneficial health effects. The monomers (+)-catechin and (−)-epicatechin and the dimers procyanidin B1 and procyanidin B2 have been identified as the main flavanols in cocoa beans. Among the flavan-3-ols, the major one is (−)-epicatechin, and among the procyanidins, its dimer B2, ranging from 0.4 to 4.5 mg/g DW and 0.3 to 10 mg/g DW, respectively, depending on the geographic origin [6]. At much lower concentrations, (+)-catechin and procyanidin B1 have also been identified in cocoa, ranging from 0.2 to 0.7 mg/g DW and 0.003 to 0.05 mg/g DW, respectively. In addition, trimers and even decamers of flavan-3-ols have also been identified in cocoa and dark chocolate [7,8].

The contents of (poly)phenols in cocoa beans depends on factors, such as variety (genotype) and origin, as well as post-harvest treatments [5,9,10]. The technological process related to the processing of cocoa beans for chocolate manufacturing affect the flavanol profile, both qualitative and quantitative [5,11]. Chocolate-making consists of a multistep process. The cocoa beans are cleaned and separated prior to industrial processing. Afterwards, they are roasted; a critical stage in the final product, since on the one hand guarantees the safety of the product and on the other hand the development of the cocoa flavors. But, it should also be noted that the high of roasting temperatures and time, lead to the loss of bioactive compounds of interest, such as (poly)phenols. Therefore, the optimization of the process to limit the loss of (poly)phenols can be of great importance for cocoa industry that wants to develop a healthy cocoa product, ensuring the safety and quality of the final product. Hence, several studies have been carried out to develop procedures to reduce the loss of phenolic compounds because there are many process that can significantly reduce the (poly)phenol content in cocoa [7,12,13]. It is known that the preservation of polyphenols during cocoa manufacture is important to the beneficial effects on human health associated with the consumption of cocoa and derived products. In a previous study, the process for obtaining a (poly)phenol enriched cocoa powder was described [14]. In this new process, some traditional stages, such as fermentation and roasting, were avoided and a step to inactivate the enzyme polyphenol oxidase was included, which helps preserve the polyphenol content in the raw cocoa bean.

The optimization of the industrial process can increase the percentage of (poly)phenols in the final products, such as cocoa powder, enabling them to be used as functional ingredients [15,16]. Several studies have shown that extracts from different cocoa beans and cocoa liquor have a powerful antioxidant activity, provided by the presence of flavonoids [8,17,18].

Many studies have described the benefits associated with the consumption of cocoa and its derivatives and this has increased the interest in obtaining functional cocoa products. As mentioned above, the benefits for human health depend not only on the amount of (poly)phenols consumed, but also on the bioavailability of the bioactive compounds [19]. In this regard, many studies have reported that the monomers, (+)-catechin and (−)-epicatechin, and the dimer procyanidin B2 are more bioavailable, being more beneficial than other flavanols with a greater molecular size [20]. The aim of this study was to evaluate the improvement in the flavanols profile and in the antioxidant capacity of the dark chocolate when using a (poly)phenol enriched cocoa powder, with the intention of yielding a functional product with potential beneficial effects for human health.

## 2. Materials and Methods

### 2.1. Chemical Reagents and Standards

All chemicals were purchased from Sigma Aldrich (St. Louis, MO, USA) while HPLC-grade solvents were purchased from either Scharlab (Barcelona, Spain) or Merck (Darmstadt, Germany). Standards of (+)-catechin and (−)-epicatechin (Sigma Aldrich, St. Louis, MO, USA) and procyanidin B1 and procyanidin B2 (Extrasynthese, Genay, France) were used for quantitative determinations.

### 2.2. Development of (Poly)Phenol Enriched Dark Chocolate and Conventional Dark Chocolate

**Figure 1:**
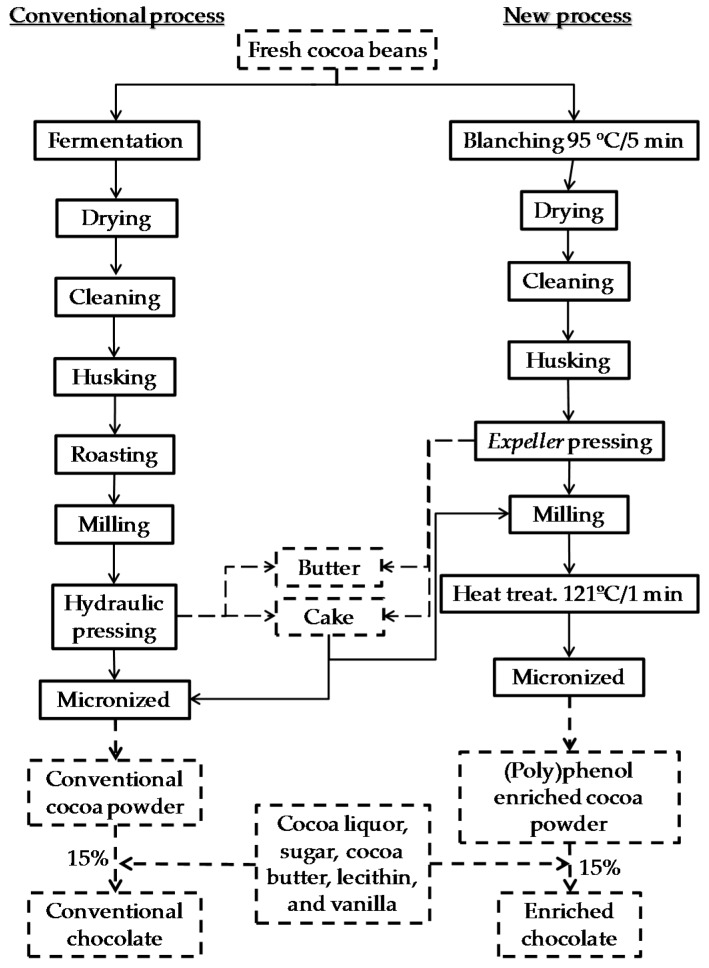
Diagram of the conventional and the new production process of (poly)phenol enriched cocoa powder [21] and chocolate.

Batches of 4 kg of (poly)phenol enriched chocolate and 4 kg of conventional dark chocolate were produced on a pilot scale (Figure 1). For the production of both chocolates, cocoa liquor (58%), sugar (20.5%), cocoa butter (6%), lecithin (0.45%), and vanillin (0.05%) were used, adding 15% (poly)phenol enriched cocoa powder for the enriched chocolate and 15% conventional cocoa powder for the conventional chocolate. The enriched cocoa powder was produced on an industrial scale from unfermented, blanched, and non-roasted cocoa beans (Amazonic-Trinitary variety-CCN51 clone from Ecuador) using the procedure described by Cienfuegos-Jovellanos et al. [21]. Briefly, a blanching treatment with water (95 °C, 5 min) was applied to fresh cocoa beans, after removal of the pulp. This enriched cocoa cake was defatted by expeller pressing up to a fat content of 11%, and micronized to give a particle size of less than 75 microns. The conventional cocoa powder (Granada brand) was a commercial cocoa powder supplied by Natra Cacao S.L. (Valencia, Spain). It was produced from fermented and dried cocoa beans (Forastero variety from East Africa), being husked, roasted, defatted by hydraulic pressing up to a fat content of 11%, and micronized to a particle size of less than 75 microns. Both cocoa powders, the enriched and the conventional, had a moisture content of 5% and a pH of 5.6. The percentage of cocoa powder (15%) added was because in the sensory test no great differences were found between the enriched chocolate and the conventional one. This was confirmed by an internal tasting panel where a group of volunteers tasted different versions of chocolates with various percentages of enriched cocoa powder, and, apparently, the one with 15% added did not seem different from the standard chocolate and was acceptable in terms of overall sensory quality.

The chocolate manufacturing process involved four steps: mixing/kneading, refining, conching, and tempering. In the first step, the ingredients (cocoa liquor, sugar, cocoa powder, and cocoa butter) were mixed together and kneaded, using refining or grinding procedures to provide a smooth chocolate paste. Then, the chocolate was subjected to the conching process, being mechanically kneaded to give it a more complete, homogeneous aroma and improved rheological characteristics. This step was carried out at temperatures between 75 °C and 80 °C, and other components—such as vanillin and lecithin—were added. Finally, the liquid chocolate was tempered, by cooling and heating under controlled conditions, and placed in molds.

### 2.3. Determination of the Antioxidant Capacity

The antioxidant capacity in cocoa powder and chocolate samples was determined by the Oxygen Radical Absorbance Capacity (ORAC) method, according to Cao et al. [22]. This method is based on the inhibition of the oxidation induced by a peroxy-radical, using a standard with antioxidant capacity as the substrate and a fluorescent probe to measure the signal. Fluorescein was used as the indicator, Trolox as the standard, and 2,2′-azobis(2-amidino-propane) dihydrochloride (AAPH) as the peroxyl radical generator. The assay was carried out using a fluorescent microplate reader (Synergy 2 Multi-Mode Microplate Reader, Biotek, Winooski, VT, USA) and 96-well black microplates equipped with a fluorescence filter having an excitation wavelength of 485 nm and an emission wavelength of 520 nm. For each calibration solution, the blank (0.075 M phosphate buffer, pH 7.0) and the samples were added to the corresponding wells. The plate reader also has an incubator and two injection pumps, which added the fluorescein and the AAPH during the assay; the temperature of the incubator was set to 37 °C. The fluorescence of each well was measured every 60 s for 90 min. The results were calculated using the standard curve provided by the instrument and expressed as µmol of Trolox Equivalents per g (µmol TE/g).

### 2.4. Analysis of Flavanols by High Performance Liquid Chromatography with UV-Vis Detection (HPLC-DAD)

The analysis of (+)-catechin and (−)-epicatechin monomers, as well as the dimers procyanidin B1 and procyanidin B2, in cocoa powder and chocolate samples was carried out by HPLC-DAD according to the method described by Andrés-Lacueva et al. [23], with some modifications. The chocolate samples were melted at 40 °C before defatting of the sample by the Soxhlet method, using petroleum ether at 55 °C for 24 h, and then drying of the sample at 40 °C. The defatted sample was homogenized in a vortex mixer with 5 mL of distilled water at 100 °C for 1 min. Twenty milliliters of methanol acidified with 0.1% HCl were added and agitated in a vortex for 2 min. Then, the homogenate was centrifuged at 1600*g* for 15 min, at 4 °C. This procedure was repeated twice, and the supernatants were combined and evaporated at 35 °C under vacuum to remove the methanol. The remaining aqueous extract was finally filtered through a 0.45-µm PTFE filter and analyzed by HPLC-DAD. The Agilent 1100 HPLC system was fitted with a quaternary pump, a degasser, a thermostatted column support, an autosampler, and a serial diode detector (Agilent Technologies, Waldbronn, GermanySpain). The separation of the different monomers and dimers was performed using a C18 Zorbax Eclipse XDB reverse phase column (150 × 2.1 mm, i.d. 5 µm) (Agilent Technologies, Spain) thermostatted at 35 °C. The mobile phases used were 0.1% aqueous formic acid (solvent A) and acetonitrile (B), at a flow rate of 0.6 mL/min. Elution began with a gradient from 4 to 10% B in 25 min, followed by a gradient to 13% B at 30 min, to 15% B at 33 min, and to 50% B at 35 min, followed by washing and then a return to the initial conditions at 45 min. Chromatograms were recorded at 280 nm. The identification of flavanols was performed by comparison of their retention times with those of the authentic standards of (+)-catechin hydrate (−)-epicatechin, procyanidin B1 and procyanidin B2. Quantification was based on calibration curves constructed using 5 to 25 ppm (+)-catechin, 20 to 100 ppm (−)-epicatechin, 1 to 10 ppm procyanidin B1, and 5 to 50 ppm procyanidin B2.

### 2.5. Analysis of Flavan-3-ols and Procyanidins by High Performance Liquid Chromatography and Fluorescence Detection (HPLC-FL)

The analysis of procyanidins in the cocoa powder and chocolate samples by HPLC-FL was carried out according to the method described by Gu et al. [8]. This included defatting of the sample (2 g) using 45 mL of n-hexane, followed by centrifugation and evaporation. After that, the extraction of the procyanidins was performed according to the methodology of Gu et al. [24]. Briefly, defatted samples were homogenized in a vortex mixer with acetone/water/acetic acid (70/29.5/0.5, *v/v/v*), sonicated at 37 °C for 15 min, and finally centrifuged at 3000 *g* for 5 min at 4 °C. The extraction process was performed twice more, each time adding 15 mL of the acetone/water/acetic acid (70/29.5/0.5, *v/v/v*) to the remnant. Finally, the supernatants obtained in each extraction stage were combined and the organic phase was removed under vacuum. The aqueous phase remaining was shaken in an ultrasonic bath and purified using a vacuum-equipped solid phase extraction unit (SPE) (Merck, Darmstadt, Germany). A lipophilic filler of Sephadex LH-20 (Scharlab, Spain) was used as the solid phase. For each sample, 3 g of Sephadex LH-20 were packed into a column (6 × 1.5 cm) and preconditioned with 15 mL of methanol/water (30/70, *v/v*) overnight before use. The sample was passed through sorbent and collected. Once the procyanidins had been retained in the adsorbent, the column was washed with 40 mL of a mixture of methanol/water (30/70, *v/v*) to remove the sugars and other phenols. The procyanidins were then recovered with 80 mL of acetone/water (70/30, *v/v*) until the cartridge was completely clean. Finally, the acetone was evaporated at 45 °C under vacuum. The aqueous extract obtained was lyophilized and dissolved in 10 mL of acetone/water/acetic acid (70/29.5/0.5, *v/v/v*), before being filtered and injected into the HPLC-FL system. The different procyanidin fractions were separated, according to their degree of polymerization, in the same Agilent 1100 HPLC system described above. Separation of the different fractions was achieved on a C-8 normal phase column, Luna Silica 100 Å (150 × 4.6mm, i.d. 5 µm) (Phenomenex, Madrid, Spain). The mobile phase used was a mixture of dichloromethane (A), methanol (B), and glacial acetic acid/water (50/50, *v/v*) (C) at a flow rate of 0.5 mL/min. Elution began with a linear gradient from 14 to 28% B in 30 min, followed by a linear gradient from 28 to 39% B at 45 min, and from 39 to 86% B at 50 min. From 50 to 55 min an isocratic gradient of 86% B was used, followed by washing and then a return to the initial conditions at 70 min. A presence of 4% C remained constant throughout the elution. A fluorescence detector, set at an excitation wavelength of 276 nm and an emission wavelength of 316 nm, was used for detection. Due to the lack of commercially available standards, the quantification of the different procyanidin fractions was performed using previously published relative response factors for each one [25]. Briefly, the response factor of (−)-epicatechin was measured based on a calibration curve constructed using a commercial standard. Then, the response factors of the different procyanidin fractions were estimated using the corresponding relative response factors (0.65 for dimers, 0.69 for trimers, 0.61 for tetramers, 0.58 for pentamers, 0.45 for hexamers, 0.62 for heptamers, 0.52 for octamers, 0.36 for nonamers, 0.56 for decamers, and 0.45 for >decamers), which were used to calculate the concentration (mg/g) of each fraction.

### 2.6. Statistical Analysis

All analytical parameters were determined in triplicate for each sample, except the procyanidins analysis by HPLC-FL which was determined in duplicated. The data were expressed as means ± standard deviations (SD). Independent-samples T-tests were applied to determine the differences between means for all analyzed parameters (*p* < 0.05). The statistical analysis was carried out using GraphPad Prism version 6.02 for Windows, GraphPad Software (La Jolla, CA, USA).

## 3. Results

### 3.1. Antioxidant Capacity

**Figure 2:**
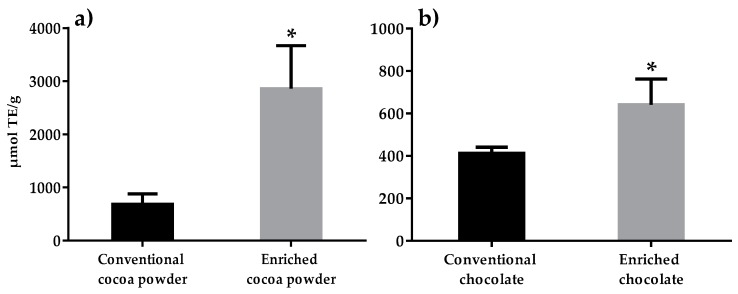
Oxygen Radical Absorbance Capacity (ORAC) antioxidant capacity of conventional and enriched cocoa powder (**a**) and chocolate (**b**). * Indicates significant differences at *p* < 0.05.

The ORAC method gave values of 686 µmol TE/g for the conventional cocoa powder and 2861 µmol TE/g (more than 4-times higher) for the (poly)phenol enriched cocoa powder. A value of 412 µmol TE/g was obtained for the conventional dark chocolate and a value of 641 µmol TE/g (1.6-times higher) was obtained for the (poly)phenol enriched chocolate (Figure 2).

### 3.2. Flavanol Analysis by HPLC-DAD

**Figure 3:**
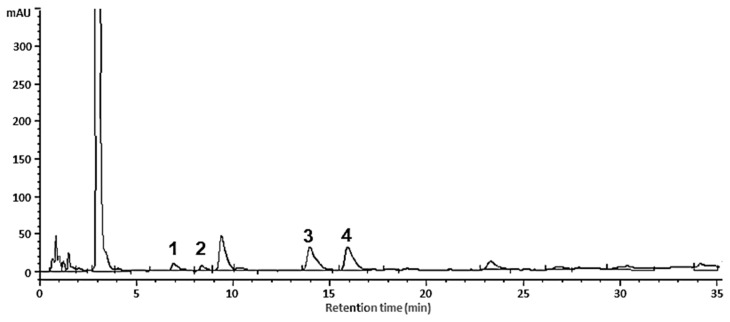
HPLC-DAD chromatogram at 280 nm of dark (poly)phenol enriched chocolate. 1: Procyanidin B1; 2: (+)-catechin; 3: procyanidin B2; 4: (−)-epicatechin.

The qualitative and quantitative profiles of cocoa powder and dark chocolate (enriched and conventional) were analyzed by HPLC-DAD. The chromatogram obtained for the dark (poly)phenols enriched chocolate (Figure 3) provides evidence that in all cases the separation and quantification of (−)-epicatechin and (+)-catechin monomers and procyanidin B2 and B1 polymers were achieved.

The results obtained for the cocoa powder and chocolate (conventional and enriched) are shown in Table 1. In both samples (cocoa powder and chocolate) the main flavanols found were (−)-epicatechin and procyanidin B2.

Total monomers content was 12-fold higher in the enriched cocoa powder than that quantified in the conventional one, while the total dimers content was 15-fold higher. In the enriched chocolate, the total monomers content was 3-fold higher than in the conventional, while the total dimers content was 2.4-fold higher. Moreover, the total content of monomers and dimers was 3.9 mg/g for the conventional chocolate and 10.0 mg/g (2.6-times greater) for the enriched chocolate (Table 1).

### 3.3. Procyanidins Analysis by HPLC-FL

**Figure 4:**
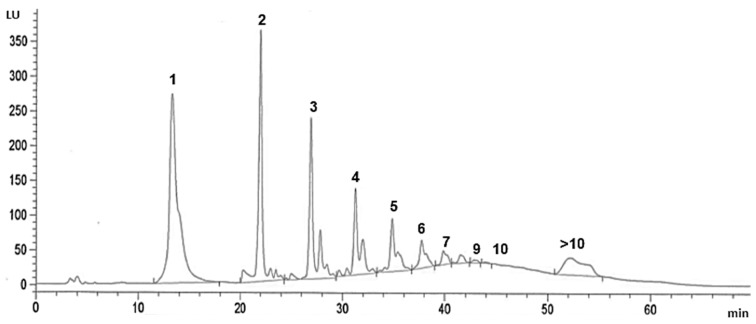
HPLC-FL chromatogram of (poly)phenol enriched cocoa (poly)phenol enriched chocolate (b) 1: monomers; 2: dimers; 3: trimers; 4: tetramers; 5: pentamers; 6: hexamers; 7: heptamers; 8: octamers; 9: nonamers; 10: decamers; >10: procyanidins with a degree of polymerization of more than 10 units.

Due to the absence of standards for some of the different fractions of procyanidins, their quantification was performed using relative response factors previously published for chocolate [25]. Figure 4 show the chromatograms obtained for the (poly)phenol enriched chocolate. The method used allowed the separation of the different fractions, from monomers to decamers; the procyanidins of a higher degree of polymerization (>10) were quantified at the end of the chromatogram as a single peak.

Table 2 shows the flavanol contents, covering the range from monomers to polymers, in the conventional and (poly)phenol enriched cocoa powder and in the conventional and enriched chocolate. In both the (poly)phenol enriched cocoa powder and enriched chocolate, the monomers were the main fraction, representing 23% of the total flavanols. However, in the conventional cocoa powder and conventional chocolate, the main fraction was comprised of the procyanidins with a degree of polymerization of more than 10 units (>10), this fraction representing 37% and 22%, respectively, of the total flavanols. Overall, the content of procyanidins in the enriched cocoa powder was 4.7-fold greater than in the conventional cocoa powder, and in the enriched chocolate it was 1.4-fold greater than in the conventional chocolate.

## 4. Discussion

A dark chocolate rich in (poly)phenols has been produced using an enriched cocoa powder, whose new production process reduces the losses of bioactive compounds, allowing these compounds to be preserved during chocolate production. The difference between the enriched and conventional cocoa powders lies in the processing, since the enriched cocoa powder was obtained from a new process in which some stages of the traditional process were replaced, such as fermentation, grain roasting, and defatting by hydraulic pressing. Conversely, other common processes used in the food industry were incorporated to minimize the loss of phenolic compounds, such as scalding to inactivate the enzyme (poly)phenol oxidase (PPO), deffating by expellers at low and controlled temperatures, and heat treatment by steam currents in an autoclave (Figure 1). The dose of cocoa powder added (15%) for the manufacturing of the chocolate samples was chosen on the grounds of a sensory test, in order to obtain no perceivable differences between the enriched chocolate and the conventional one. In spite of the astringency and the purple color of the enriched cocoa powder, this dose did not change the organoleptic properties of the enriched chocolate. Moreover, the enriched cocoa powder had a very plain profile, with no cocoa taste or aroma, since it had not undergone the roasting process; therefore, the precursors of the cocoa aroma and flavor were not developed since it was not fermented either.

The antioxidant capacity of the enriched cocoa powder used for the production of the dark chocolate rich in (poly)phenols was 2861 µmol TE/g representing 317% higher than that of the conventional cocoa powder. This shows the enhanced value of this (poly)phenol enriched cocoa powder. The antioxidant capacity of the enriched chocolate was 641 µmol TE/g representing 56% higher than that of the conventional chocolate, but both values are significantly higher than the corresponding values found in various fruit powders—acai (400 µmol TE/g), blueberry (260 µmol TE/g), cranberry (310 µmol TE/g), and pomegranate (190 µmol TE/g)—and in natural, non-alkalized cocoa powder (634 µmol TE/g) [17].

Taking into account that the chocolates were elaborated with 15% cocoa powder (enriched or conventional), theoretically, the antioxidant capacity should be 103 µmol TE/g for the conventional chocolate and 429 µmol TE/g for the enriched one. However, the values obtained were 4-fold higher for the conventional chocolate (412 µmol TE/g) and 1.5-fold higher for the enriched one (641 µmol TE/g). The differences observed between the hypothetical and the analytical results could be explained, in part, by the presence of other bioactive compounds in the cocoa liquor added (58%) during the chocolate manufacturing [5], which could increase the antioxidant capacity in the chocolate. In addition, the results obtained demonstrate that the enriched powder is less stable than the conventional one during chocolate processing. However, the antioxidant capacity of the enriched chocolate formulation, including 15% (poly)phenols enriched cocoa powder, was increased by 56% compared to the chocolate formulated with 15% conventional cocoa powder.

To achieve beneficial effects on human health, the daily recommended intake of antioxidants is 3000–3600 µmol TE [26]. However, the recommended five pieces of fruit and vegetables per day do not reach 50% of this value, providing between 1200 and 1640 µmol TE [26]. For this reason, the consumption of functional products enriched in (poly)phenols could be a strategy to increase the daily intake of antioxidants. Specifically, a 10-g portion of the enriched dark chocolate described here will provide a high level of antioxidants, more than 6000 µmol TE.

The analysis of the enriched chocolate measured by HPLC-DAD, showed that the total flavan-3-ols content was 4 mg/g representing a 186% higher than that of the conventional chocolate. Moreover, the content in enriched chocolate was also higher than that determined by other authors in dark chocolate with values ranging to 0.24–0.45 mg/g [27]. The results showing that, for chocolate, (−)-epicatechin was the second most abundant of the flavanols analyzed agree with other work reporting that one of the main compounds in cocoa beans is (−)-epicatechin [28]. In this study, the sum of the monomers, (−)-epicatechin and (+)-catechin, in the enriched chocolate (4.0 mg/g) was higher compared to the conventional one (1.4 mg/g), and was higher than that obtained by other authors for a dark chocolate (1.9 mg/g) [8]. These results show that, in the new functional product, there is an increase in the more bioavailable flavanols and therefore in the health benefits of the dark (poly)phenol enriched chocolate, compared to the conventional chocolate. This is because (+)- catechin, (−)-epicatechin and procyanidin B2, which represent 43% of the total procyanidins, are more bioavailable than the other compounds, as some authors have shown, since their absorption in the gastrointestinal tract is higher [20,29,30,31,32].

The total flavanol content, covering the range from monomers to polymers, of the enriched cocoa powder was 78 mg/g representing 364% higher than that obtained for the conventional cocoa powder in this study and 90% higher than that obtained for a conventional cocoa powder by other authors who reported a value of 41 mg/g [8]. The mean contents quantified for the flavan-3-ols and procyanidins in this study are similar to the values published for a cocoa powder that was not affected by post-harvest variables [12,33]. Similarly, it should be noted that the total procyanidins content quantified in the (poly)phenol enriched cocoa powder was 1.3-fold higher than that quantified by Kealey et al. [12] in a cocoa powder obtained from fresh unfermented beans, lyophilized and defatted by Soxhlet, and 2.8-fold greater than that quantified by Misnawi et al. [33] in a cocoa powder obtained from fresh unfermented beans partially defatted using an expeller.

Gu et al. [8], using a similar method of extraction, purification with Sephadex LH20, and normal phase analysis using HPLC-ESI/MS with a fluorescence detector, reported values of total procyanidins from 8.5 to 20 mg/g in conventional dark chocolate samples - very similar to the 15 mg/g for conventional chocolate and the 21 mg/g in enriched chocolate found in this study. In addition, the total procyanidin value of 21 mg/g in cocoa powder samples recorded by Miller et al. [34] is close to that found here for conventional cocoa powder (17 mg/g). However, the (poly)phenol enriched chocolate developed in the present study has also been characterized by values of low-molecular-weight procyanidins higher than previously published data ranging values to 1.14–1.77 mg/g [8,35]. The sum of the classes ranging from monomers to hexamers found in the enriched chocolate was 17 mg/g, compared to 10 mg/g found by Gu et al. [8] in a dark chocolate and 11 mg/g found in this work for conventional chocolate. Theoretically, as both chocolates (enriched and conventional) were elaborated with 15% cocoa powder, the total flavanol content should be 11.7 mg/g for the enriched chocolate and 2.5 mg/g for the conventional, 2-fold and 12-fold higher than that quantified for the enriched chocolate and for the conventional one, respectively. These results could be explained, in part, by the presence of flavanols in the cocoa liquor added (58%) during the chocolate manufacturing.

## 5. Conclusions

Our results describing the formulation of a new dark chocolate enriched with (−)-epicatechin and procyanidin B2 was successfully achieved, as well as a notable enrichment of the oligomeric procyanidin fractions (from monomers to hexamers), when compared with a conventional dark chocolate. These results are of current interest to both large food companies and health professionals. The manufacturing of the enriched dark chocolate following the procedures described above would allow the use of a health claim related to cocoa flavanols. According to the Commission Regulation (EU) 2015/539 of 31 March 2015 [36], the new enriched chocolate could have the claim ‘*Cocoa flavanols help maintain the elasticity of blood vessels, which contributes to normal blood flow*’. In order to obtain this beneficial effect, 10 g of the new enriched dark chocolate should be consumed daily, which would provide more than 200 mg of total flavanols (flavan-3-ols and procyanidins ranging from dimers to decamers). In addition, the new dark chocolate formulation, including 15% (poly)phenols enriched cocoa powder, increased greatly the antioxidant properties compared to a conventional dark chocolate, while maintaining the organoleptic properties unchanged. However, a future consumer preference study could be of great interest to determine the acceptability of the new functional chocolate.

## Figures and Tables

**Table 1:** Flavanol contents (mg/g) in the conventional and enriched dark chocolate analyzed by reverse phase HPLC-DAD ^1^.

Flavanols	Conventional Cocoa Powder	Enriched Cocoa Powder	Conventional Chocolate	Enriched Chocolate
(+)-catechin	0.5 ± 0.1	3.4 ± 0.0 *	0.3 ± 0.0	0.6 ± 0.0 ***
(**−**)-epicatechin	1.9 ± 0.5	24.9 ± 0.0 ***	1.1 ± 0.1	3.4 ± 0.2 ***
Procyanidin B1	0.1 ± 0.0	2.1 ± 0.1 ***	1.2 ± 0.1	1.8 ± 0.1 **
Procyanidin B2	1.0 ± 0.2	14.2 ± 0.3 ***	1.3 ± 0.2	4.2 ± 0.1 ***
Total	3.5 ± 0.8	44.6 ± 0.3 ***	3.9 ± 0.1	10.0 ± 0.1 ***

^1^ The values are the average of three replicates (*n* = 3) ± SD. *, **, *** Indicates significant differences at *p* < 0.05, *p* < 0.01 and *p* < 0.001, respectively, between conventional and enriched for each products (cocoa powder and chocolate).

**Table 2:** Flavanol contents (mg/g) of the conventional and enriched cocoa powder and chocolate analyzed by normal phase HPLC-FL ^a^.

Flavanols	Conventional Cocoa Powder	Enriched Cocoa Powder	Conventional Chocolate	Enriched Chocolate
Monomers	2.70 ± 0.20	17.70 ± 0.10 ***	3.20 ± 0.09	4.70 ± 0.10 **
Dimers	3.00 ± 0.20	16.70 ± 1.40 **	2.90 ± 0.20	4.20 ± 0.30 *
Trimers	2.00 ± 0.20	13.00 ± 0.90 **	2.00 ± 0.01	3.20 ± 0.20 *
Tetramers	1.30 ± 0.08	10.90 ± 0.07 ***	1.50 ± 0.02	2.30 ± 0.10 **
Pentamers	0.77 ± 0.00	7.00 ± 0.06 ***	0.97 ± 0.01	1.50 ± 0.00 ***
Hexamers	0.51 ± 0.00	5.00 ± 0.40 **	0.66 ± 0.01	1.00 ± 0.02 **
Heptamers	0.13 ± 0.00	1.70 ± 0.30 *	0.20 ± 0.01	0.30 ± 0.03 *
Octamers	0.12 ± 0.02	0.90 ± 0.10 **	0.15 ± 0.01	0.20 ± 0.04
Nonamers	0.04 ± 0.01	0.47 ± 0.02 **	0.08 ± 0.02	0.14 ± 0.02
Decamers	nd	0.10 ± 0.10	nd	0.02 ± 0.01
>Decamers	6.20 ± 0.90	4.57 ± 0.19	3.30 ± 0.30	3.20 ± 1.10
Total	16.8 ± 1.6	78.0 ± 1.5 *	15.0 ± 0.5	20.8 ± 0.9 *

^a^ The values are the average of two replicates (*n* = 2) ± SD. nd (not detected). *, **, *** Indicates significant differences at *p* < 0.05, *p* < 0.01, and *p* < 0.001, respectively, between conventional and enriched for each product (cocoa powder and chocolate).
